# Efficacy and safety of neoadjuvant immunotherapy combined with chemotherapy in locally advanced esophageal cancer: A meta-analysis

**DOI:** 10.3389/fonc.2022.974684

**Published:** 2022-09-05

**Authors:** Jincheng Wang, Kun Zhang, Tianzhou Liu, Ying Song, Peiyan Hua, Shu Chen, Jindong Li, Yang Liu, Yinghao Zhao

**Affiliations:** ^1^ Department of Thoracic Surgery, The Second Hospital of Jilin University, Changchun, China; ^2^ Jilin Provincial Key Laboratory on Molecular and Chemical Genetics, The Second Hospital of Jilin University, Changchun, China; ^3^ Department of the Gastrointestinal Surgery, The Second Hospital of Jilin University, Changchun, China; ^4^ Gastroenteric Medicine and Digestive Endoscopy Center, The Second Hospital of Jilin University, Changchun, China

**Keywords:** neoadjuvant immunotherapy, locally advanced esophageal carcinoma, efficacy, safety, meta-analysis

## Abstract

**Objective:**

The progress of neoadjuvant therapy for resectable locally advanced esophageal cancer has been stagnant. There has been much progress in immunotherapy for advanced esophageal cancer, but the efficacy and safety of neoadjuvant immunotherapy for resectable locally advanced esophageal cancer have not yet been definitively demonstrated.

**Methods:**

Original articles describing the safety and efficacy of neoadjuvant immunotherapy in resectable locally advanced esophagus published until July 2022 were retrieved from PubMed, Embase, and the Cochrane Library. The ratio (OR) and 95% confidence interval (CI) were calculated to conduct heterogeneity and subgroup analysis.

**Results:**

In total, 759 patients from 21 studies were enrolled. The effectiveness of neoadjuvant immunotherapy in combination with chemotherapy was evaluated using the major pathologic response (MPR) and pathologic complete response (PCR). In the enrolled patients, 677 were treated surgically and 664 achieved R0 resection. Major pathological remission was achieved in 52.0% (95% CI: 0.44–0.57) of patients on neoadjuvant immunotherapy combined with chemotherapy and complete pathological remission in 29.5% (95% CI: 0.25–0.32) of patients. The safety was primarily assessed by the incidence of treatment-related adverse events (TRAEs) and surgical resection rates. The incidence of TRAEs and the surgical resection rate combined ORs were 0.15 (95% CI: 0.09–0.22) and 0.86 (95% CI: 0.83–0.89), respectively.

**Conclusion:**

Neoadjuvant immunotherapy combined with chemotherapy in locally advanced resectable esophageal cancer is effective and safe.

## 1 Background

Esophageal cancer, with more than 500,000 new cases diagnosed each year, was the seventh most common cancer and the sixth leading cause of cancer death worldwide in 2018 (1). Esophageal cancer may be divided into two broad histological subtypes: esophageal squamous cell carcinoma and esophageal adenocarcinoma. The worldwide most common histological subtype in ESCC accounts for 87% of all esophageal cancers (2). Esophagectomy remains the mainstay of treatment for esophageal cancer. Nevertheless, amongst patients with locally advanced esophageal cancer, surgery alone often has a high rate of recurrence and metastasis. Chemotherapy has been recommended by some guidelines as the first neoadjuvant treatment for these patients (3, 4). At best, neoadjuvant chemotherapy increased the R0 resection rate by 6% and the 5-year survival rate by 5.9% (5, 6).

In most Western countries, neoadjuvant radiotherapy (nCRT) plus surgery has been chosen as the standard of care for patients with locally advanced esophageal cancer, based on the results of the CROSS trial (7). It was demonstrated that while neoadjuvant radiotherapy can further improve the R0 resection rates, it is associated with more postoperative complications and higher postoperative mortality (8, 9). Therefore, a new more effective and less toxin-inducing neoadjuvant regimen is warranted to enhance clinical outcomes in esophageal cancer patients with no increase in the occurrence of treatment-related adverse events (TRAEs).

Programmed cell death protein 1 (PD-1) is expressed by activated lymphocytes, and by binding to ligands including programmed cell death ligand 1 (PD-L1). It blocks the immune response and promotes immune escape, further contributing to the development of various malignancies and disease progression (10, 11). PD-1 inhibitors, which block the PD-1/PD-L1 linkage as a novel immunotherapeutic tool, have been widely used in many tumors (10, 12). The appropriate combination of chemotherapeutic agents with PD-1 blockers may enhance the efficacy of PD-1 blockers, in particular for tumors that are weakly immunogenic and have poor chemotherapy sensitivity (13). The combination of PD-1 inhibitors with chemotherapy has been shown in preclinical studies to further enhance the host immune response and inhibit the immune escape of cancer cells (14). Additionally, a recent combination of nivolumab and pembrolizumab chemotherapy for neoadjuvant treatment in locally advanced ESCC has demonstrated acceptable treatment response, progression-free survival (PFS), and overall survival (OS) (15). The immunotherapy significantly improved the 5-year survival rate of advanced ESCC in the KEYNOTE and ATTRACTION studies (16, 17).

Multiple study meta-analyses would offer more optimistic options for several neoadjuvant treatment tactics and gain the confidence for future clinical trials of neoadjuvant immunotherapy. The aim of this meta-analysis, based on available data, is to demonstrate the efficacy and safety of neoadjuvant immunotherapy combined with chemotherapy in locally advanced esophageal cancer and to provide further treatment options for future locally advanced esophageal cancer with better survival benefits. Until now, there has been no published meta-analysis on similar topics.

## 2 Materials and methods

### 2.1 Study control

We independently carried out the search, data analysis, and writing. No other person was involved. The trial protocol can be found in PROSPERO under the registration number CRD42022331592.

### 2.2 Search strategy and study selection

Comprehensive English language searches were conducted using PubMed, Embase, and the Cochrane Library to find published articles on neoadjuvant immunotherapy for locally advanced esophageal cancer reported up to July 1, 2022. We also retrieved the most recent unpublished data on ongoing clinical trials of neoadjuvant immunotherapy for locally advanced esophageal cancer at international oncology congresses such as ASCO and ESMO up to July 1, 2022. Medical Subject Headings are used to search for terms such as esophageal cancer, neoadjuvant therapy and immunotherapy (including all currently known ICIs). Please refer to the supplementary files for a detailed search strategy. The reference lists of all full texts retrieved were screened to further identify potentially relevant studies.

### 2.3 Selection criteria and data extraction

The publications that fulfilled the following criteria were selected: 1. the publication reported resectable locally advanced esophageal cancer; 2. the ICIs were presently utilized in clinical practice or registered clinical trials; and 3. reports include full regimens, patient data, and at least one key clinical outcome, such as MPR, PCR, the incidence of TRAEs, and surgical resection rates. Publications were excluded if the following criteria were met: 1. the existence of inoperable or metastatic disease; 2. the focus of the study was not on MPR, PCR, TRAE incidence, or surgical resection rates; 3. there were fewer than 10 patients included; 4. validated data to evaluate the efficacy and safety of neoadjuvant immunotherapy in combination with chemotherapy were lacking; 5. there were duplicate publications; and 6. in breach of any of the above inclusion criteria. Two researchers (WJC and LY) independently reviewed each of the retrieved publications. After review by the senior researcher (ZYH), discrepancies between the two reviewers were resolved through discussion and consensus. The full text of relevant articles was then searched to assess their eligibility. Citations were also manually reviewed for relevant reports to identify additional studies.

### 2.4 Statistical analysis

Meta-analysis was performed using Review Manager version 5.4 (RevMan; (Cochrane Collaboration), which is a specialist software provided by the Cochrane Collaboration (18). As most of the included studies were single-arm clinical trials with MPR and PCR as the primary outcome indicators, the research team performed a meta-analysis using non-comparative binary data in RevMan software. The p-values and standard error (SE(p)) were calculated according to the following formula: p = ln(odds) = ln(X/(n-x)). SE(p) = SE(ln(odds)) = √1/X+1 (n-x). The odds ratio (OR) and 95% confidence interval (CI) were the efficacy measures. Heterogeneity was identified with the use of the χ^2^ test and the I^2^ test. To determine that the joint results were not heavily influenced by individual trials, the included studies were taken out in turn for sensitivity analysis. Where heterogeneity was significant, a random-effects model was used; alternatively, a fixed-effects model was used. *p* < 0.05 was considered a statistically significant difference. A Higgins I^2^ statistic of <50% was considered low heterogeneity and >50% of the statistic was considered high heterogeneity. Subgroup analysis was performed to determine the source of heterogeneity and factors associated with clinical outcomes. The data were statistically analyzed using RevMan 5.4 software and Stata/SE 15.0 software.

### 2.5 Assessments of publication bias and study quality

The quality of included studies was assessed using the Cochrane Handbook 5.1.0 recommended risk of a bias assessment tool, including (1) random allocation method; (2) allocation concealment; (3) whether to adopt a blind method for the participants and researchers; (4) whether the outcome was assessed by a blind method; (5) completeness of outcome data; (6) selective reporting of outcomes; and (7) other bias. The qualitative evaluation was carried out independently by the two researchers and differences of opinion were solved by discussion between the two or by a third researcher. Possible publication bias in clinical studies was examined by funnel plots.

## 3 Results

### 3.1 Results of search

**Figure 1:**
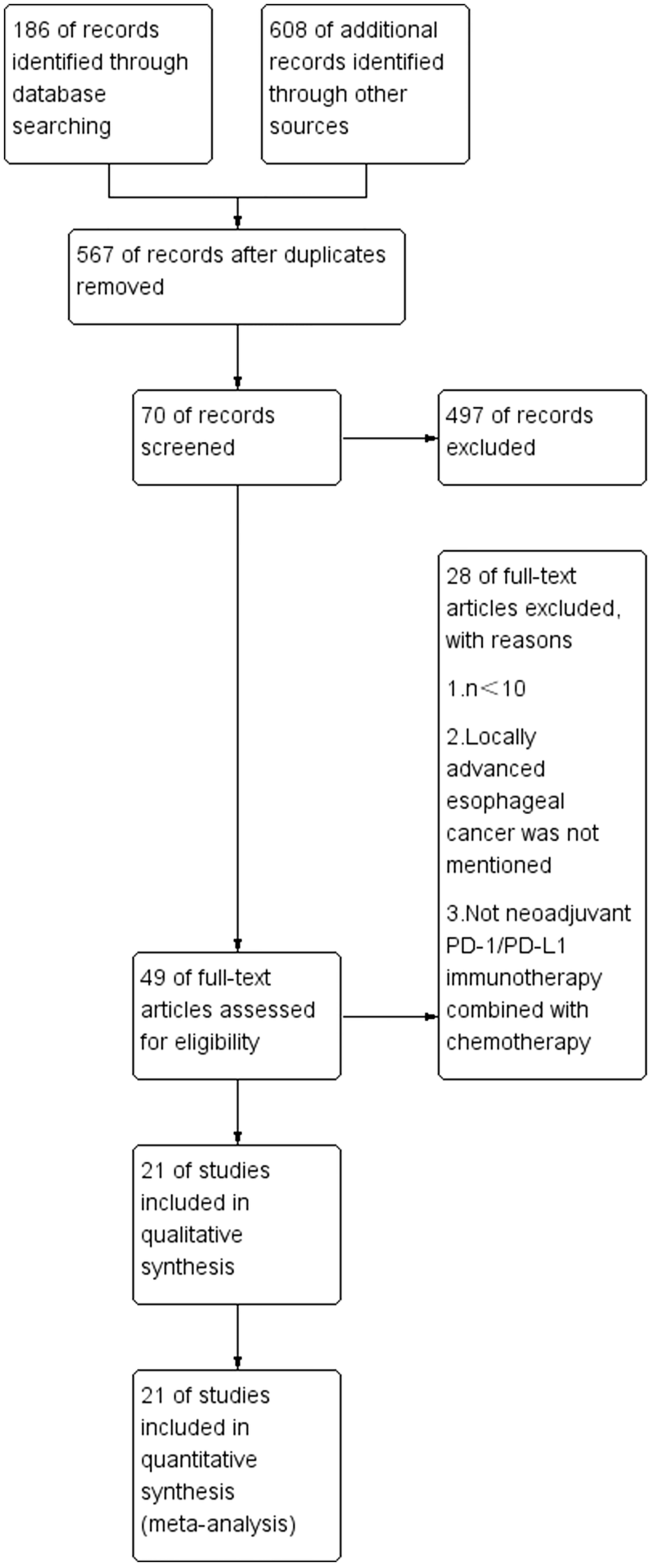
Publication search.

**Figure 2:**
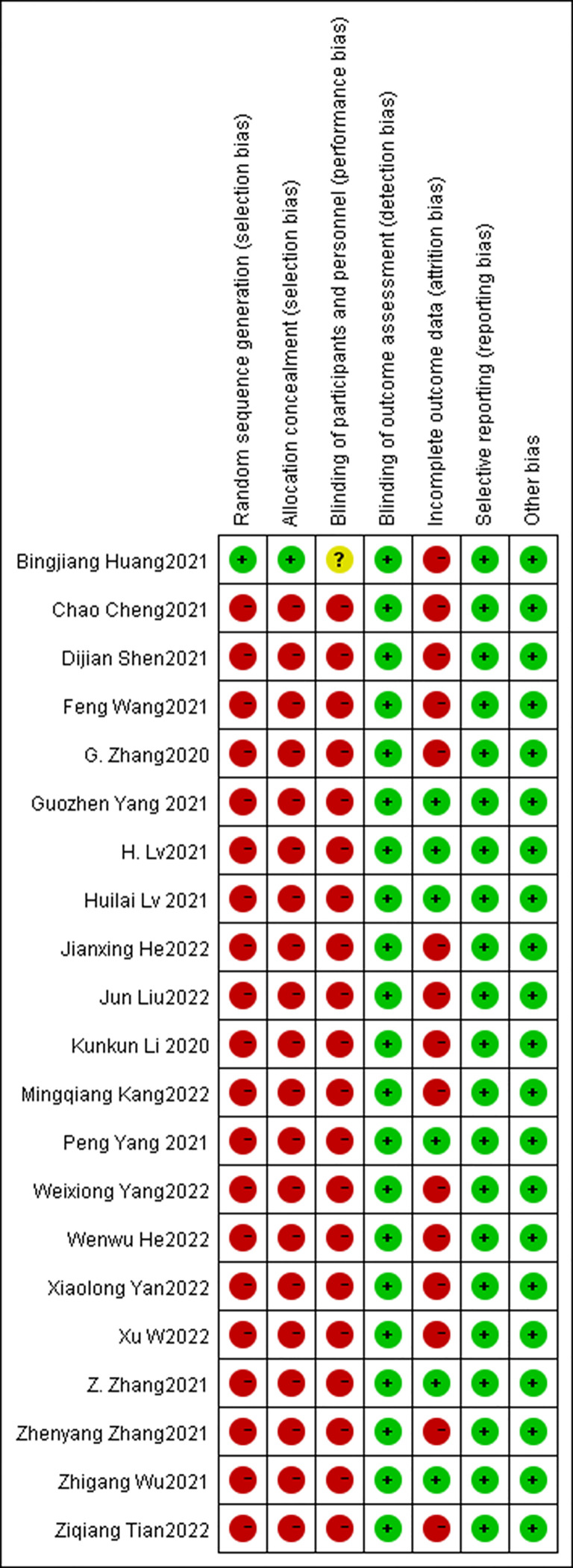
Assessments of Publication Bias and Study Quality.

In accordance with the study strategy, the first search retrieved 794 documents, 227 duplicates were removed, 567 were removed based on the title and abstract, and 70 were finally selected for a full detailed examination. A total of 49 full texts were available free of charge and after a careful reading of the full texts, 28 studies were excluded as they did not meet the inclusion criteria. In the end, 21 studies, which included 759 patients, were used for the analysis. Of the 21 studies included, there were 20 single-arm open-label cohort studies (15, 19–37) and there was one two-arm open-label randomized controlled trial (RCT) (38). Of the 21 studies included, three are still ongoing (26, 29, 30) and 18 (15, 19–25, 27, 28, 31–38) have been completed. The main neoadjuvant immunotherapy drugs include pembrolizumab, sintilimab, and camrelizumab, amongst others. **Figure 1** shows the detailed study selection process and **Figure 2** the low risk of summary bias for the included studies.

### 3.2 Patient characteristics

A total of 759 patients were enrolled in the study, 38 (24) of whom received one cycle of neoadjuvant immunotherapy combined with chemotherapy, the remaining patients received two to four cycles of neoadjuvant immunotherapy combined with chemotherapy, 677 patients underwent follow-up surgery, and 18 (26, 29, 30) patients awaited surgery. R0 resection was achieved by 664 (15, 19–38) patients. Further details of the patient characteristics are summarized in **Supplementary Table**.

### 3.3 Primary outcomes

#### 3.3.1 Efficacy of neoadjuvant immunotherapy

##### 3.3.1.1 MPR

**Figure 3:**
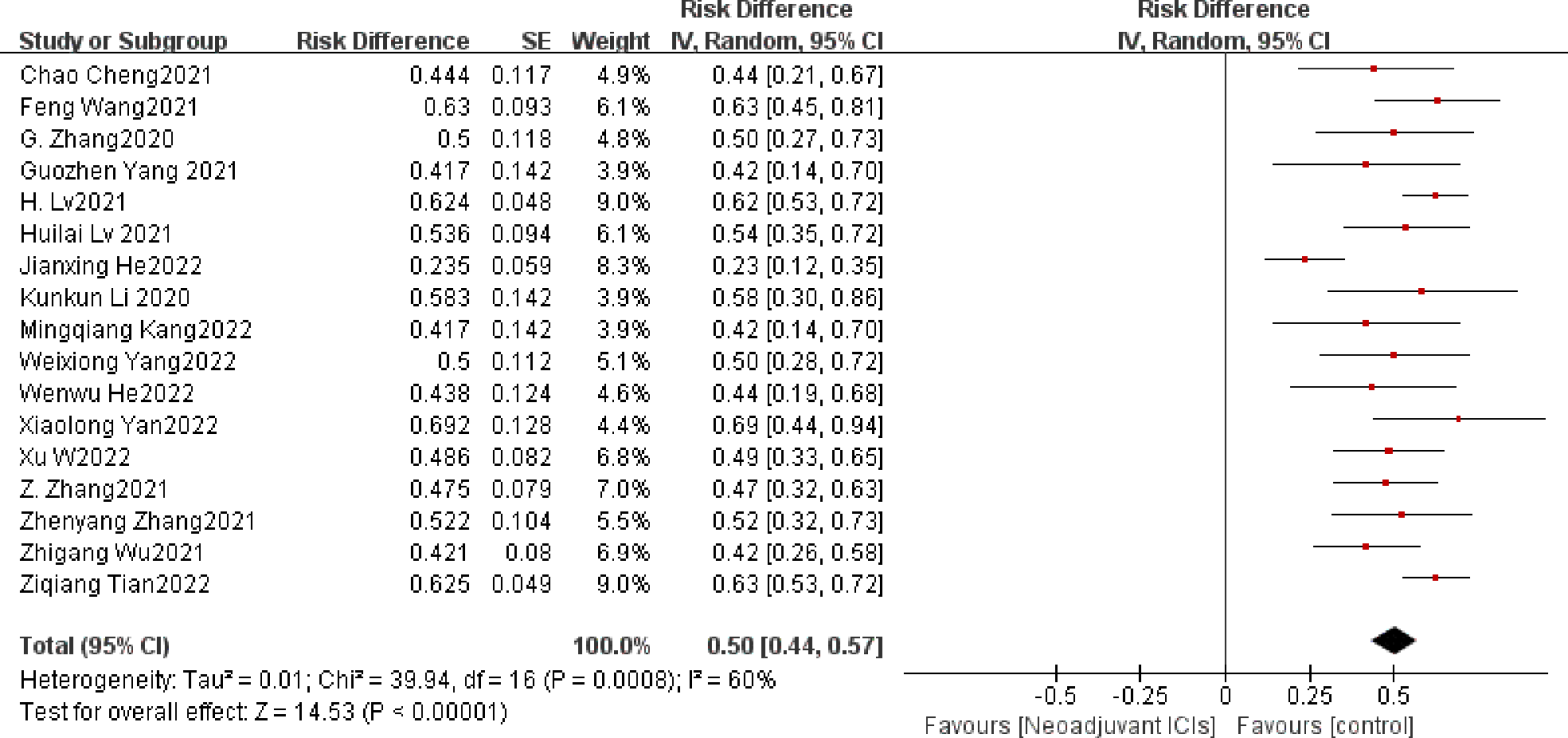
MPR neoadjuvant immunochemotherapy efficacy forest plot. By definition, an OR < 1 implies a therapeutic advantage of neoadjuvant immunochemotherapy for locally advanced esophageal cancer. MPR, a major pathological response; PCR, a pathological complete response; ICIs, immune checkpoints inhibitors; IV, inverse variance; CI, confidence interval; OR, odds ratio.

The definition of MPR is less than 10% of the remaining viable tumor cells in the resected primary tumor. The average MPR was 50.3%. Each of the 17 trials had an individual OR that supported neoadjuvant immunotherapy in combination with chemotherapy (individual OR < 1.0) (20–22, 24–37). Based on the 17 included studies above, the combined MPR showed statistically significant differences (OR=0.50; 95% CI, 0.44–0.57; P=0.0008; **Figure 3**). Because heterogeneity was statistically significant, a random-effects model was applied (*p*<0.001, I^2 =^ 60%).

##### 3.3.1.2 PCR

**Figure 4:**
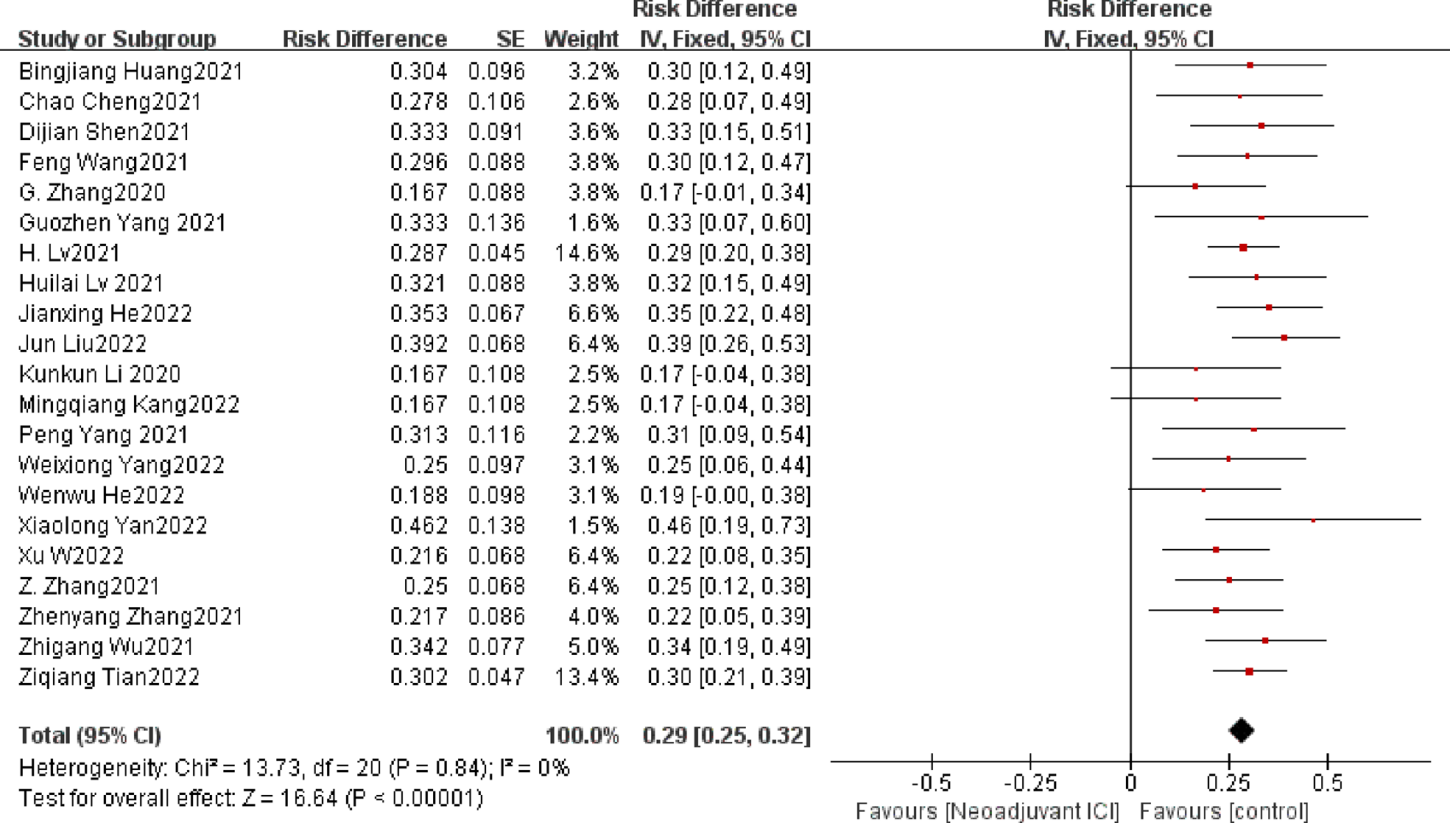
PCR neoadjuvant immunochemotherapy efficacy forest plot.

Another common and powerful predictor of the efficacy of neoadjuvant therapy is PCR, often defined as the absence of viable tumor cells. In the 21 qualifying experiments, the average PCR amounted to 28.3%. Individual ORs in each eligible study supported neoadjuvant immunotherapy in combination with chemotherapy (individual OR < 1.0) (15, 19–38). The combined OR was 0.29 (95% CI, 0.25–0.32), a statistically significant difference (*p* < 0.001) that overall favored neoadjuvant immunotherapy in combination with chemotherapy. A fixed-effects model was used because no significant heterogeneity was found between the 21 studies (P= 0.84, I^2^ = 0%; **Figure 4**).

##### 3.3.1.3 R0 resection rate

**Figure 5:**
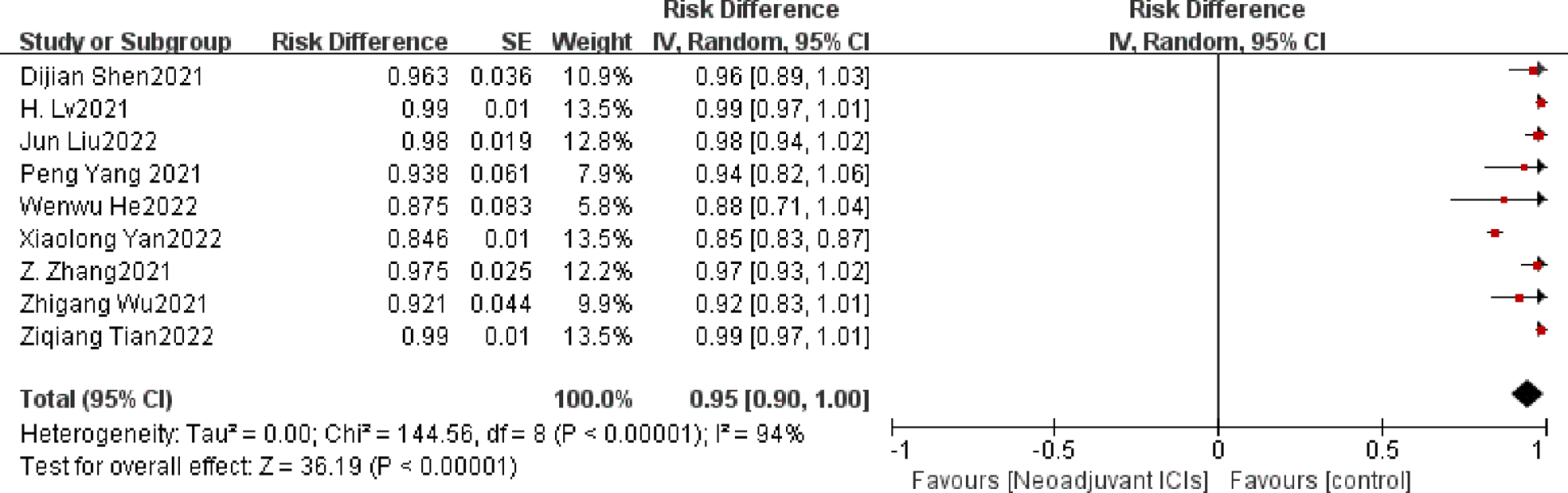
R0 resection rate neoadjuvant immunochemotherapy efficacy forest plot.

R0 resection rate was defined as a complete resection of the tumor with a negative microscopic edge, meaning that no tumor remained. It is also another important indicator to evaluate the effectiveness of neoadjuvant therapy. Twelve of the 21 studies achieved 100% R0 resection, with an overall mean R0 resection rate of 97.5%. The remaining nine studies (15, 19, 23–25, 27, 32, 36, 37) had individual ORs as well as combined ORs <1 (OR=0.95,95% CI: 0.90–1.00, *p*<0.001; **Figure 5**). Heterogeneity (*p*<0.001, I^2 =^ 94%) was significant, so a random-effects model was used.

#### 3.3.2 Safety of neoadjuvant immunotherapy

##### 3.3.2.1 The incidence of TRAE

**Figure 6:**
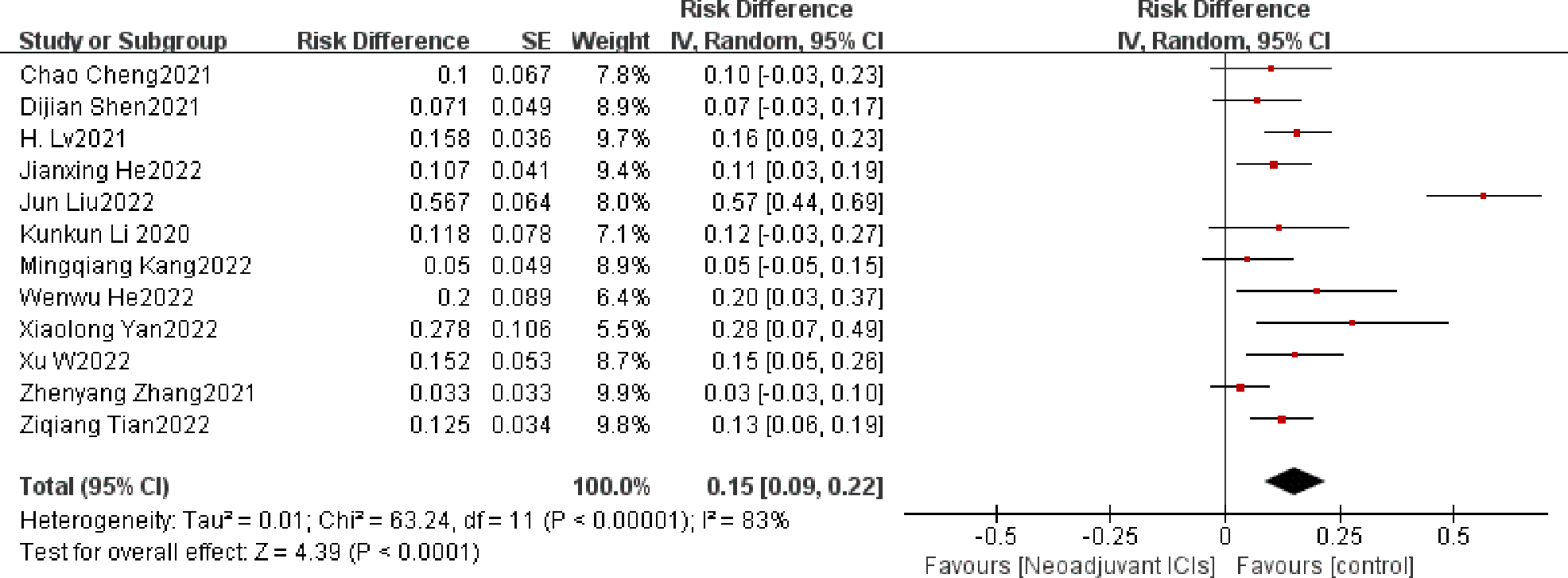
TRAEs incidence neoadjuvant immunotherapy chemotherapy forest plot.

The occurrence of TRAE is to be defined as an adverse event caused by ICIs, which is assessed by the National Cancer Institute Common Terminology Criteria for Adverse Events (NCI-CTCAE) version 4; it is a key metric for evaluating the security of neoadjuvant immunotherapy (39). A total of twelve (15, 20, 23, 25, 28, 30, 32–37) of the included clinical studies reported the incidence of grade 3 or higher TRAE in a total of 512 patients. The overall average incidence of TRAE was 16.3%. Overall individual ORs and summary analysis indicated support for neoadjuvant immunotherapy in combination with chemotherapy, with an individual OR of below 1 and a composite OR of 0.15 (95% CI, 0.09–0.22). The differences were statistically significant (*p*<0.001; **Figure 6**). As the heterogeneity was significant (*p* < 0.001, I^2 =^ 83%), a random-effects model would have been appropriate. Grade 3–5 TRAEs comprised a single case of death due to pneumonia and acute respiratory failure (23) and a solitary case of death due to immune-related pneumonia (30). The others were mainly manageable adverse events such as myelosuppression, leukopenia, neutropenia, anemia, fatigue, and thrombocytopenia, which did not contribute to serious adverse outcomes or result in higher postsurgical rates of mortality.

##### 3.3.2.2 Surgical resection rate

**Figure 7:**
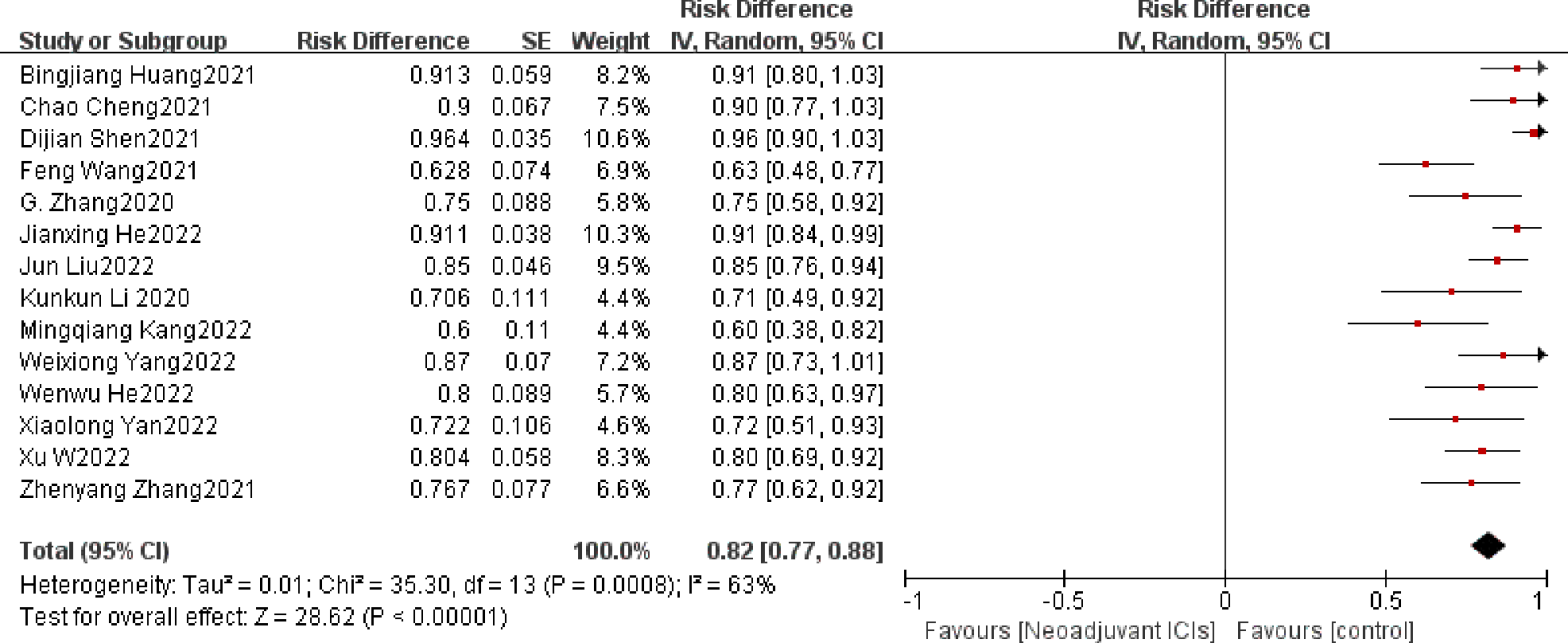
Surgical resection rate neoadjuvant immunotherapy chemotherapy forest plot.

The surgical resection rate represents the ratio of surgically resected patients to those expected to be resected and it is also an important indicator for evaluating the safety of neoadjuvant immunotherapy (39). In seven (19, 21, 22, 24, 27, 32, 37) of the 21 studies, the surgical resection rate was 100%. The overall average surgical resection rate was 86.6%. There is clear support for neoadjuvant immunotherapy in combination with chemotherapy in both individual OR and pooled OR (individual OR<1.0; pooled OR=0.82; 95% CI, 0.77–0.88; *p* < 0.001; **Figure 7**). Using a random-effects model, significant heterogeneity was observed (P=0.0008, I^2 =^ 63%).

##### 3.3.2.3 Incidence of surgical complications and surgical delay rate

Another common metric for evaluating the safety of neoadjuvant immunotherapy in combination with chemotherapy is the incidence of surgical complications, defined as operation-related complications that occur during the perioperative period. The incidence of surgical complications was only mentioned in three experiments (23, 24, 37), with incidences of 47.1%, 26.3% and 51.0%. No postoperative deaths were reported and only two patients (23) developed serious cardiac complications due to supraventricular tachycardia and congestive heart failure, while other complications included anastomotic fistula, pleural effusion, pneumonia, pulmonary infection, postoperative hemorrhage, and postoperative hoarseness, most of which had a good prognosis. The other common metric used to assess the safety of neoadjuvant immunotherapy in combination with chemotherapy is the rate of surgical delay; usually, the ratio of patients delayed in surgery due to adverse events caused by neoadjuvant immunotherapy to all patients expected to have surgery. Of the 21 studies included, only five mentioned (20–22, 28, 34) no surgical delays. In the study by Jun Liu et al. (23), eight patients had delayed surgery due to trade, and the median time to delayed surgery was 19 days (range 7–48).

### 3.4 Sensitivity analysis

**Figure 8:**
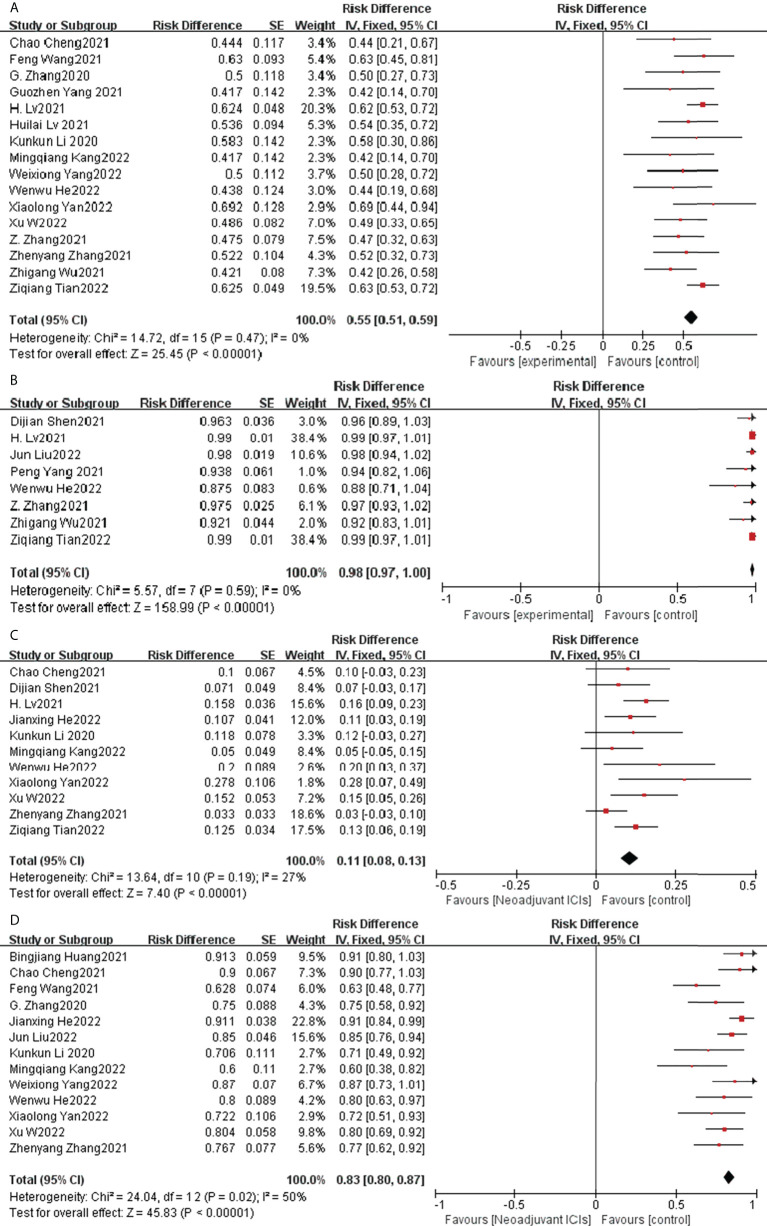
Sensitivity analysis of the incidence of MPR **(A)**, R0 resection rates **(B)**, TRAEs **(C)**, surgical resection rate **(D)**.

Rechecking the search, choice, and incorporation criteria for studies did not reduce heterogeneity. To ascertain that the joint results were not heavily influenced by individual trials, the included studies were taken out in turn for sensitivity analysis. In an analysis of individual studies of MPR incidence in 17 studies, the study by Jianxing He et al. (35) was the most important factor for heterogeneity, although it did not carry the greatest weight in the study. Excluding the significant reduction in heterogeneity after this study (P= 0.47; I^2^ = 0%), the combined results of the remaining 16 trials still significantly demonstrated the safety of neoadjuvant immunotherapy in combination with chemotherapy (OR = 0.55; 95% CI: 0.51 -0.59; *p* < 0.001; **Figure 8A**). As well, the study by Xiaolong Yan et al. (36) was the main reason for the heterogeneity of R0 resection rates. After removal, the combined OR of the remaining eight trials was 0.98 (95% CI: 0.97-1.00; **Figure 8B**), which still supports neoadjuvant immunotherapy in combination with chemotherapy (*p* < 0.001).

In an individual study analysis of TRAE incidence across the twelve studies, the JunLiu et al. (23) study was the most significant contributor to heterogeneity, although it did not account for the greatest weighting in the study. Excluding the significant reduction in heterogeneity after this study (P= 0.19; I^2^ = 27%), the combined results of the remaining eleven trials still significantly demonstrated the safety of neoadjuvant immunotherapy in combination with chemotherapy (OR = 0.11; 95% CI, 0.08 –0.13; *p* < 0.001; **Figure 8C**). In a similar vein, the study by Dijian Shen et al. (15) contributed most to the heterogeneity of surgical resection rates. After removal, the remaining nine trials had a combined OR of 0.83 (95% CI, 0.80–0.87; **Figure 8D**), which still supports neoadjuvant immunotherapy in combination with chemotherapy (*p* < 0.001). Collectively, the results of the sensitivity analysis continue to support the efficacy and safety of neoadjuvant immunotherapy in combination with chemotherapy.

### 3.5 Exploratory subgroup analysis

**Figure 9:**
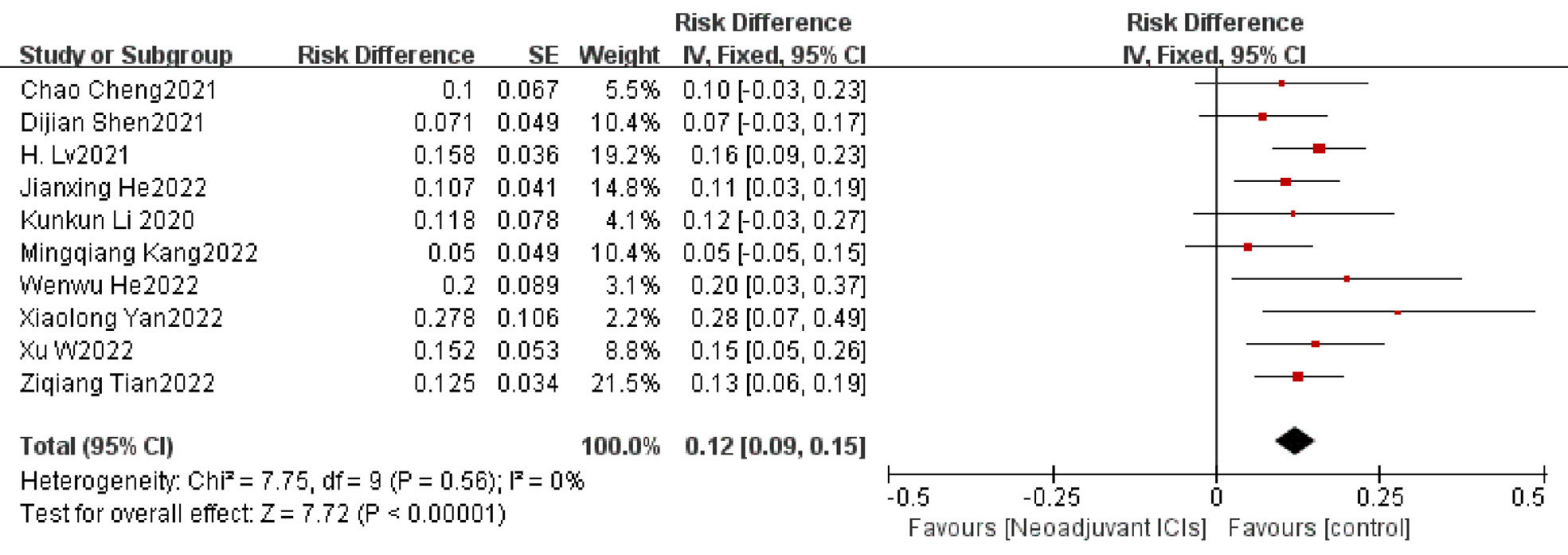
Subgroup analysis of the incidence of TRAEs.

**Figure 10:**
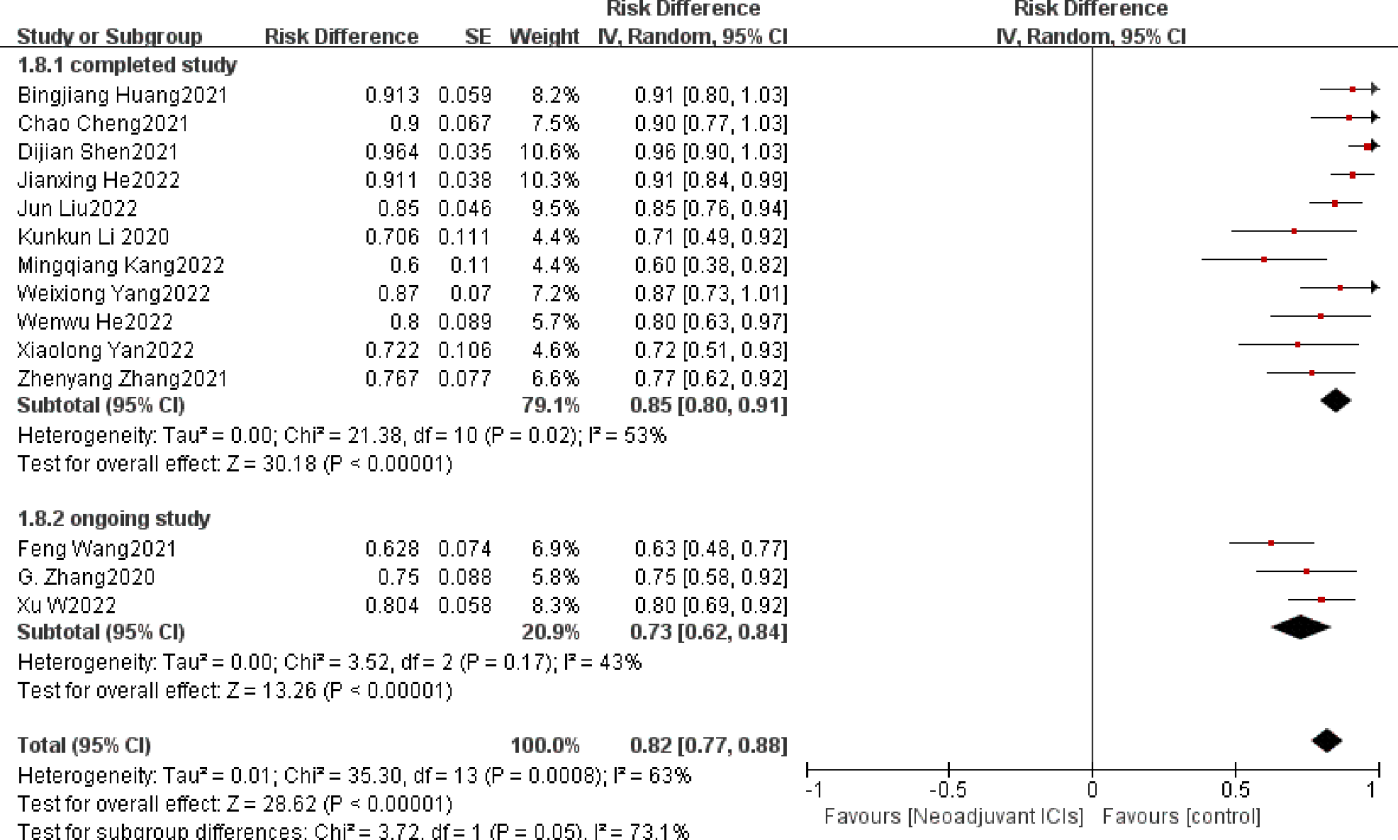
Subgroup analysis of the surgical resection rate.

**Figure 11:**
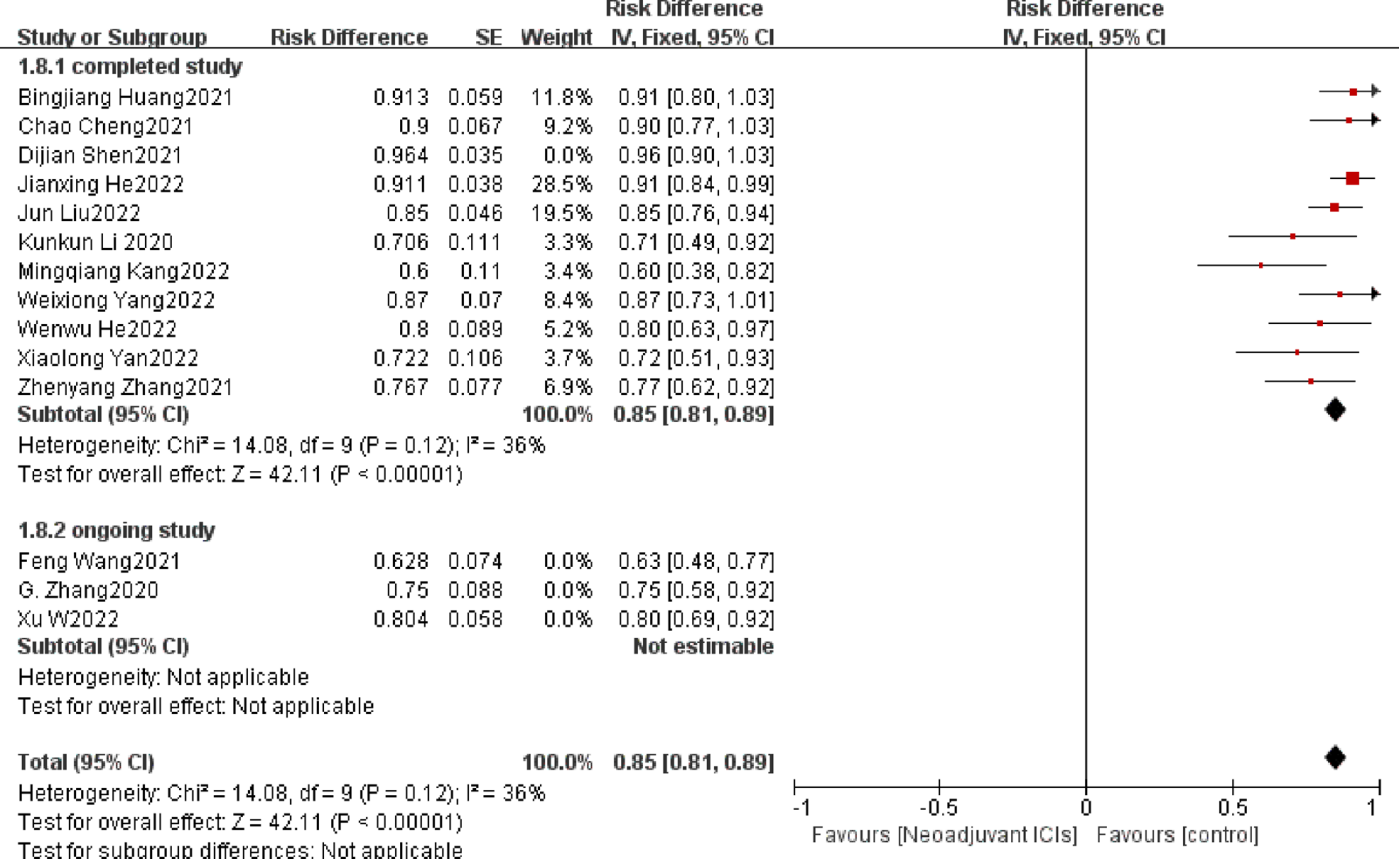
Sensitivity analysis was performed again after subgroup analysis of resection rate.

A subgroup analysis was performed by random grouping to further identify possible sources of heterogeneity. Seventeen studies reported the incidence of MPR and nine studies reported R0 resection rates, and after excluding studies with large heterogeneity, I^2 =^ 0%, so no subgroup analysis was required. Twelve studies reported the incidence of TRAEs, with I^2 =^ 27% after excluding studies with greater heterogeneity, and then further subgroup analysis. Heterogeneity decreased significantly after removing the study by Zhenyang Zhang (33) et al. (OR=0.12, 95%CI: 0.09-0.15; P=0.56; I^2 =^ 0% **Figure 9**). The subgroup analysis of surgical resection rates distinguished completed studies from incomplete studies, taking into account that three studies (26, 29, 30) were still ongoing, but heterogeneity not decreased significantly (P=0.02, I^2 =^ 53%; **Figure 10**). With further sensitivity analysis, heterogeneity decreased significantly after removing the Dijian Shen et al. (15) study (P=0.12; I^2 =^ 36%; **Figure 11**).

**Figure 12:**
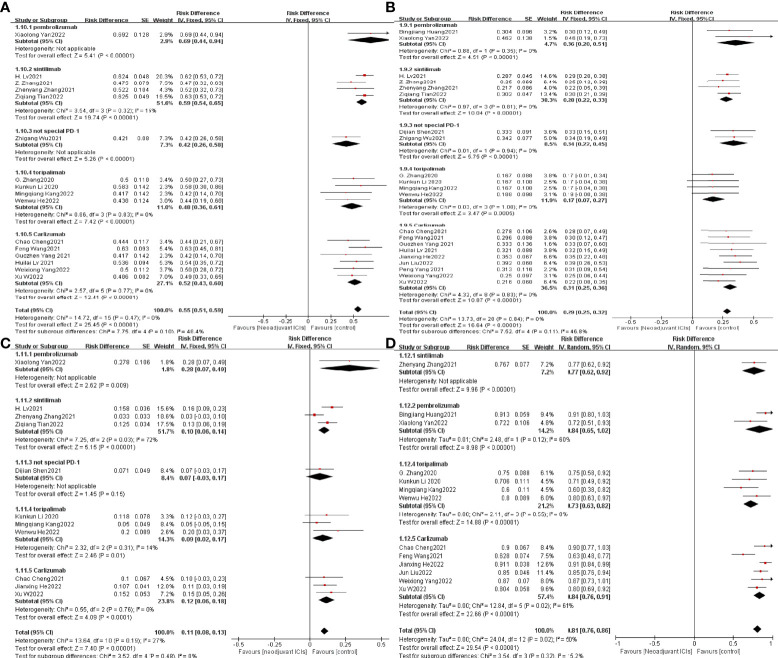
Subgroup analysis was based on ICIs categories grouped separately with MPR **(A)**, PCR **(B)**, TRAEs **(C)**, and surgical resection rates **(D)**.

To understand the possible correlation between the type of ICI and the outcome of neoadjuvant immunotherapy in combination with chemotherapy, we performed subgroup analysis. The 21 selected studies included two studies in which patients were treated with pembrolizumab (36, 38), four studies in which patients were treated with sintilimab (27, 32, 33, 37), four studies in which patients were treated with toripalimab (20, 25, 26, 34), and nine studies in which patients were treated with Carlizumab (19, 21–23, 28–31, 35).The potential correlation between ICI type and the efficacy and safety of neoadjuvant immunotherapy in combination with chemotherapy has not seen significant findings, implying that ICI type does not currently predominate in neoadjuvant immunotherapy (**Figure 12**).

### 3.6 Publication bias test

**Figure 13:**
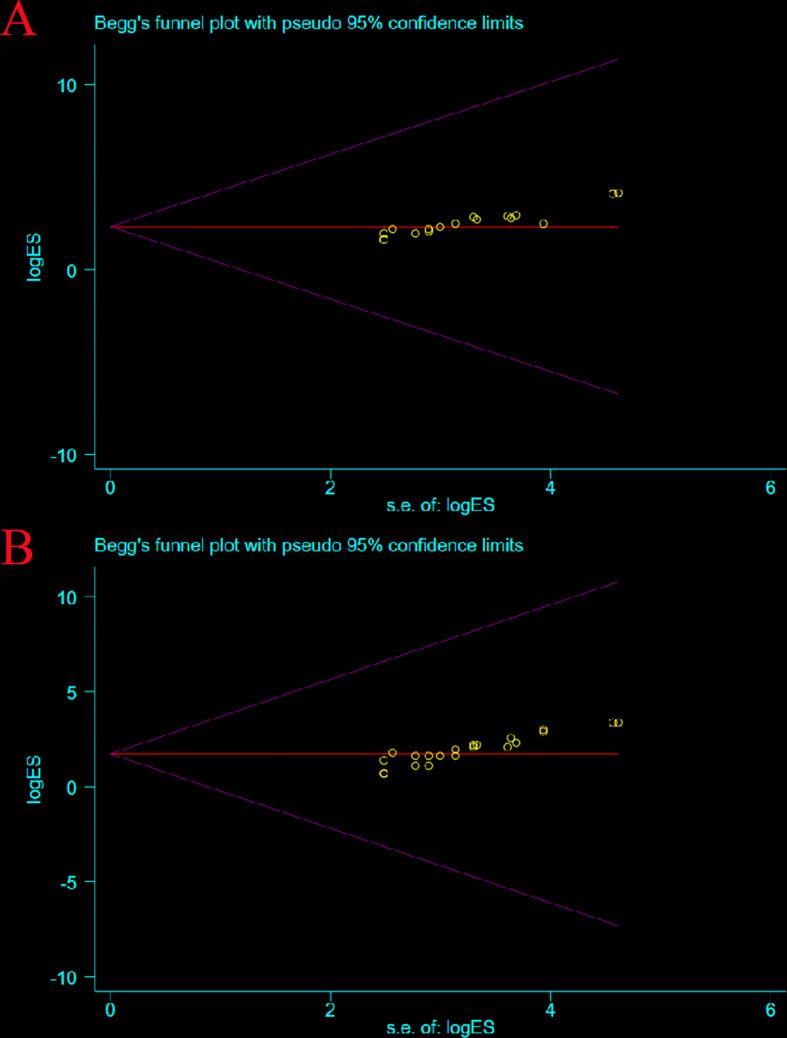
Publication Bias Test.: MPR **(A)**; pCR **(B)**.

The possible publication bias in 21 clinical studies was examined by funnel plots in the analysis of the efficacy and safety of neoadjuvant immunotherapy in combination with chemotherapy. Since most of the data collected were single-arm clinical trials with no controls, the images showed asymmetric funnel plot distributions without significant publication bias, although Pr > |z| = 0.000 and P > |t| = 0.000 for MPR and PCR (**Figure 13**).

## 4 Discussion

During the perioperative treatment, chemotherapy plus radiotherapy based on platinum represents the current treatment approach. Chemotherapy with radiotherapy represents the current standard of care and has been adopted as a combination partner for immunotherapy in many countries and several ongoing trials (4, 40). The safety issues associated with radiotherapy, though, prompted the search for a less toxic treatment option (41–43).

At this point, neoadjuvant immunotherapy in combination with chemotherapy remains controversial, but our study supports the effectiveness and safety of neoadjuvant immunotherapy in combination with chemotherapy in locally advanced esophageal cancer. The combination of neoadjuvant immunotherapy with chemotherapy had a mean PCR of 28.3% in our meta-analysis. Ten (15, 19, 21–24, 35–38) of the 21 studies had a PCR of more than 30%, with the highest PCR of 46.2% in the study by Xiaolong Yan (36) and colleagues, which is higher than the previously reported neoadjuvant chemotherapy (10.2%) (44). Astoundingly, among the 17 included clinical studies, the mean MPR was 50.3%, with the highest MPR of 69.2% in the clinical trial by Xiaolong Yan et al. (36). Five studies (20, 29, 32, 36, 37) had MPRs greater than 55%. In the KEYNOTE-181 trial of neoadjuvant chemotherapy, postoperative pathology in 18 evaluable patients revealed MPR in nine (50%) (16). This is similar to the mean MPR rate of our included studies. With these encouraging results, we provide sufficient evidence for the effectiveness of neoadjuvant immunotherapy in combination with chemotherapy.

In terms of surgical safety, the mean R0 resection rate of neoadjuvant immunotherapy combined with chemotherapy was 97.5%. In previous studies, the R0 resection rates for neoadjuvant chemotherapy and neoadjuvant radiotherapy were 60% and 98%, respectively (5, 45). This is another indication that the effect of neoadjuvant immunotherapy in combination with chemotherapy is encouraging and appealing. However, it is difficult to state the benefit of neoadjuvant immunotherapy in combination with chemotherapy for prolonged survival because of the short follow-up period and the fact that complete survival data have not been published. Only data from Peng Yang et al. (19) showed a 12-month progression-free survival (PFS) of 83% with a median follow-up of 18.3 months (95% CI: 16.2–20.5) and a 1-year OS of 90.9% with a median follow-up of 19.2 months (95% CI: 17.7–20.7). Future OS and PFS data are expected to shed light on the long-term situation and the benefit of neoadjuvant immunotherapy in combination with chemotherapy on survival.

It is also important to note that the safety analysis indicates that neoadjuvant immunotherapy can be continued with confidence. In our meta-analysis, the mean incidence of grade ≥3 TRAEs was 16.3%, which demonstrated good tolerability and was lower than the incidence of ≥ grade 3 TRAEs (39.5%) in neoadjuvant chemotherapy in the ESCORT study (46). The mortality rate of TRAEs is low, with only two deaths (23, 30) associated with the pneumonia-related disease. In addition, a majority of ICIs had been previously assessed in complete preliminary clinical studies and had been used in the treatment of a variety of advanced tumors and therefore there is extensive experience in the identification and treatment of adverse events. This is further evidence for the effective treatment of TRAEs. As for the surgical resection rate, the combination of neoadjuvant immunotherapy with chemotherapy had a mean level of 86.6%. The incidence of surgical complications was mentioned in only three trials [47.1% (23) and 26.3% (24) and 51.0% (37)]. No postoperative deaths were reported, and only two patients (23). It should be particularly noted that there was a case of hypersensitivity to careolizumab in the study by Feng Wang et al. (29). Given all these promising results, the safety of neoadjuvant immunotherapy in combination with chemotherapy is acceptable.

A subgroup analysis based on the type of ICI showed no evidence that different ICIs contribute differently to the efficacy and safety of neoadjuvant immunotherapy in combination with chemotherapy. Therefore, no one preferred ICI is currently available for neoadjuvant immunotherapy in combination with chemotherapy. The choice of neoadjuvant immunotherapy agents should be made on an individual patient and clinical setting basis. More clinical trial data are, of course, needed to support this conclusion.

This meta-analysis also has some limitations. First, the majority of the enrolled clinical studies have not achieved their endpoints. Consequently, a few clinical studies did not have complete protocols and data. Additionally, to the extent that most of the data came from conference abstracts, there were no official publications of these studies in these cases; therefore, bias assessment may be hampered and publication bias may be present. However, because the funnel plot for evaluating publication bias is symmetrically distributed, publication bias due to article type is acceptable. There are other indicators besides those mentioned in this paper that can be used to evaluate efficacy and safety, such as CR, PR, DCR, SD, PFS, OS, and operation time. Nevertheless, because of a lack of relevant data, we failed to use these indicators. Additional major limitations are the small sample size included in some of the experiments and the too few randomized controlled trials, which may lead to bias. Thus, there is a need for larger sample sizes and more RCTs for further validation in multicenter studies.

In locally advanced ESCC, after three cycles of treatment there is no increased toxicity of neoadjuvant immunochemotherapy (22). The metastasis of lymph nodes is highly relevant to the poor prognosis of ESCC, but a significant proportion of patients keep yp-N positive after neoadjuvant therapy, which may lead to postoperative relapse (47). A study indicated that patients with esophageal cancer who reached the descending stage after neoadjuvant therapy may have better survival outcomes (48). The data showed that patients with neoadjuvant therapy had a significantly improved quality of life and a significant relief of dysphagia symptoms, which may be associated with high PCR and stage reduction rates (31). Moreover, following this neoadjuvant treatment, the adhesion of most esophageal tumors to the surrounding tissue is looser and easier to remove, unlike after radiation therapy or neoadjuvant treatment for lung cancer (15). This encouraging clinical evidence supports the use of immunotherapy in combination with chemotherapy in neoadjuvant treatment.

While we have achieved relatively good results, there are still some issues that deserve to be looked at. The first is the number of cycles of neoadjuvant therapy. It is unknown whether this increase in the number of cycles will improve treatment efficacy, produce better MPR rates, and PCR rates, and increase toxicity and side effects; second, whether sequencing of chemotherapeutic agents with immune agents will better improve metrics such as PCR rates; third, if postoperative neoadjuvant therapy combined with chemotherapy is still needed for patients who achieve PCR; and fourth, because it is not possible for us to currently screen those patients who will benefit most from neoadjuvant immunochemotherapy treatment, predictive biomarkers are urgently needed for identification. Lastly, it is uncertain that a high postoperative PCR rate necessarily implies a high survival rate. The follow-up period of the currently included studies is too short to provide conclusive results.

In summary, there is clinical support for the widespread use of neoadjuvant immunotherapy in combination with chemotherapy as demonstrated by our meta-analysis of its efficacy and safety in locally advanced esophageal cancer. Nonetheless, long-term outcomes and toxicity must be examined to confirm this conclusion, as most clinical trials have not yet met their endpoints.

## Data availability statement

The original contributions presented in the study are included in the article/**Supplementary Material**. Further inquiries can be directed to the corresponding author.

## Author contributions

JW and YZ: Conceptualization, Methodology. JW, KZ and TL: Software, Formal analysis. JW, YL and YS: Investigation, Resources, Data Curation, extracted data from studies, and matched inclusion and exclusion criteria. JW and PH: Writing - Original Draft, Writing - Review & Editing. SC, JL and YZ: Visualization, Project administration, Supervision. All authors had full access to all data, critically revised the paper, approved the final analysis, and took responsibility for all aspects of the work to ensure that issues relating to the accuracy or integrity of any part of the work could be appropriately investigated and resolved. We warrant that the article is the original work, hasn't received prior publication.

## Funding

This study was funded by the Jilin Provincial Education Department (JJKH20201098KJ), The Health and Medicine Special Fund of Jilin Provincial Science and Technology Department (20200708116YY) and Chinese Society Of Clinical Oncology (Y-2019AZQN-0049). The funding body had no role in the design of the study and collection, analysis, and interpretation of data and in writing the manuscript.

## Conflict of interest

The authors declare that the research was conducted in the absence of any commercial or financial relationships that could be construed as a potential conflict of interest.

## Publisher’s note

All claims expressed in this article are solely those of the authors and do not necessarily represent those of their affiliated organizations, or those of the publisher, the editors and the reviewers. Any product that may be evaluated in this article, or claim that may be made by its manufacturer, is not guaranteed or endorsed by the publisher.
