# Supplementary material for: Efficacy and safety of neoadjuvant immunotherapy combined with chemotherapy in locally advanced esophageal cancer: A meta-analysis
Source: Front Oncol. 2022 Sep 5;12:974684. doi: 10.3389/fonc.2022.974684 (PMC9495441; doi:10.3389/fonc.2022.974684)
Supplement: Supplementary file 1 [file Table_1.docx]

**Supplementary File**

The search terms include medical subject terms or keywords: (((neoadjuvant or preoperative or Neoadjuvant Therapies or Therapy, Neoadjuvant or Neoadjuvant Treatment or Neoadjuvant Treatments or Neoadjuvant Systemic Therapy or Neoadjuvant Systemic Treatment or Neoadjuvant Chemotherapies or Neoadjuvant Chemotherapy Treatment) AND (Chemotherapy or Pharmacotherapy or Multimodal Treatment or Modality Therapies, Combined)) AND (Immunotherapy or Immunotherapies or pembrolizumab OR toripalimab OR avelumab OR ipilimumab OR atezolizumab OR nivolumab OR PD-1 OR PD-L1 OR CTLA-4)) AND (Esophageal cancer or Esophageal Neoplasm or Esophagus Cancer or esophageal carcinoma).

**Supplementary Table**

| **Study** | **Drug** | **Age** | **CR** | **PR** | **SD** | **PCR** | **MPR** | **R0 resection rate** |
| --- | --- | --- | --- | --- | --- | --- | --- | --- |
| Peng Yang2021 | Paclitaxel + carboplatin + carrelizumab | 60.9±7.8 | 4/16 | 9/16 | 3/16 | 5/16 | - | 15/16 |
| Kunkun Li2020 | Paclitaxel+carboplatin +toripalimab | - | - | - | - | 2/12 | 7/12 | 12/12 |
| Huilai Lv2021 | Albumin paclitaxel+cisplatin+carrizumab | 18-75 | - | - | - | 9/28 | 15/28 | 28/28 |
| Guozhen Yang  2021 | Paclitaxel +S1 capsule + carritizumab | 56 | - | 7/12 | 5/12 | 4/12 | 5/12 | 12/12 |
| Dijian Shen  2021 | Albumin paclitaxel + carboplatin +PD-1 | 62.2 | 12/27 | 12/27 | 3/27 | 9/27 | - | 26/27 |
| Jun Liu  2022 | Paclitaxel + carboplatin + carrelizumab | 65 | - | - | - | 20/51 | - | 50/51 |
| Zhigang Wu  2021 | Paclitaxel + platinum +PD-1 | 61 | - | - | - | 13/38 | 16/38 | 35/38 |
| Wenwu He  2021 | Paclitaxel + carboplatin +toripalimab | 61.4±6.5 | 6/18 | 5/18 | 7/18 | 3/16 | 7/16 | 14/16 |
| 1. Zhang2020 | Nab-p, S-1 capsule and toripalimab | - | - | 19/24 | 5/24 | 3/18 | 9/18 | 18/18 |
| Z. Zhang2021 | Paclitaxel + carboplatin +sintilimab | - | - | 33/40 | 7/40 | 10/40 | 19/40 | 39/40 |
| H. Lv2021 | Sintilimab in combination with chemotherapy | 65 | - | - | - | 29/101 | 63/101 | 100/101 |
| ChaoCheng2021 | Albumin paclitaxel + carboplatin + caririzumab | - | - | - | - | 5/18 | 8/18 | 18/18 |
| Feng Wang2021 | Docetaxel + nedaplatin + caririzumab | - | - | - | - | 8/27 | 17/27 | 27/27 |
| Xu W2022 | Albumin paclitaxel + carboplatin + caririzumab | - | - | - | - | 8/37 | 18/37 | 37/37 |
| WeixiongYang  2022 | Paclitaxel + carboplatin + carrelizumab | 58.6±10.1 | - | 3/20 | 2/20 | 5/20 | 10/20 | 20/20 |
| Bingjiang Huang2021 | Docetaxel + nedaplatin + Pembrolizumab | - | 7/23 | 13/23 | 2/23 | 7/23 | - | 21/21 |
| Zhenyang Zhang2021 | Albumin paclitaxel + cisplatin + Sintilimab | 58.3±7.1 | - | 20/30 | 9/30 | 5/23 | 12/23 | 23/23 |
| Mingqiang Kang2022 | Docetaxel + cisplatin +Toripalimab | 58.3 | - | 14/20 | 5/20 | 2/12 | 5/12 | 12/12 |
| Jianxing He2022 | Paclitaxel + cisplatin + carrizumab | 61 | 7/51 | 27/51 | 17/51 | 18/51 | 12/51 | 51/51 |
| Xiaolong Yan2022 | Pembrolizumab + Docetaxel/Nab-paclitaxel +Nedaplatin | 60.9 | 4/18 | 5/18 | - | 6/13 | 9/13 | 11/13 |
| Ziqiang Tian2022 | sintilimab + platinum + taxanes | - | - | - | - | 29/96 | 60/96 | 95/96 |
| **EVENT RATE** | - | - | 28.2% | 53.1% | 23.3% | 28.3% | 50.3% | 97.5% |

**Supplementary table**: A summary of pathological features of the locally advanced esophagus treated with neoadjuvant immunochemotherapy (CR: complete response; PR, partial response; SD, stable disease; PCR, complete pathological response; MPR, major pathological response).
